# Insulin/IGF-1 signaling promotes immunosuppression via the STAT3 pathway: impact on the aging process and age-related diseases

**DOI:** 10.1007/s00011-021-01498-3

**Published:** 2021-09-02

**Authors:** Antero Salminen, Kai Kaarniranta, Anu Kauppinen

**Affiliations:** 1 Department of Neurology, Institute of Clinical Medicine, University of Eastern Finland, P.O. Box 1627, 70211 Kuopio, Finland; 2 Department of Ophthalmology, Institute of Clinical Medicine, University of Eastern Finland, P.O. Box 1627, 70211 Kuopio, Finland; 3 Department of Ophthalmology, Kuopio University Hospital, KYS, P.O. Box 100, 70029 Kuopio, Finland; 4 School of Pharmacy, Faculty of Health Sciences, University of Eastern Finland, P.O. Box 1627, 70211 Kuopio, Finland

**Keywords:** Ageing, Alzheimer’s, FoxO, Immunosenescence, mTOR, Tolerance

## Abstract

**Background:**

The insulin/IGF-1 signaling pathway has a major role in the regulation of longevity both in *Caenorhabditis elegans* and mammalian species, i.e., reduced activity of this pathway extends lifespan, whereas increased activity accelerates the aging process. The insulin/IGF-1 pathway controls protein and energy metabolism as well as the proliferation and differentiation of insulin/IGF-1-responsive cells. Insulin/IGF-1 signaling also regulates the functions of the innate and adaptive immune systems. The purpose of this review was to elucidate whether insulin/IGF-1 signaling is linked to immunosuppressive STAT3 signaling which is known to promote the aging process.

**Methods:**

Original and review articles encompassing the connections between insulin/IGF-1 and STAT3 signaling were examined from major databases including Pubmed, Scopus, and Google Scholar.

**Results:**

The activation of insulin/IGF-1 receptors stimulates STAT3 signaling through the JAK and AKT-driven signaling pathways. STAT3 signaling is a major activator of immunosuppressive cells which are able to counteract the chronic low-grade inflammation associated with the aging process. However, the activation of STAT3 signaling stimulates a negative feedback response through the induction of SOCS factors which not only inhibit the activity of insulin/IGF-1 receptors but also that of many cytokine receptors. The inhibition of insulin/IGF-1 signaling evokes insulin resistance, a condition known to be increased with aging. STAT3 signaling also triggers the senescence of both non-immune and immune cells, especially through the activation of p53 signaling.

**Conclusions:**

Given that cellular senescence, inflammaging, and counteracting immune suppression increase with aging, this might explain why excessive insulin/IGF-1 signaling promotes the aging process.

## Highlights

The aging process is associated with chronic low-grade inflammation.

Insulin/IGF-1 signaling regulates the longevity of *C. elegans* and mammalian species.

Insulin/IGF-1 signaling stimulates STAT3 signaling through several pathways.

STAT3 signaling evokes anti-inflammatory/immunosuppressive responses

Insulin/IGF-1 signaling promotes the aging process through STAT3 signaling.

## Introduction

**Fig. 1:**
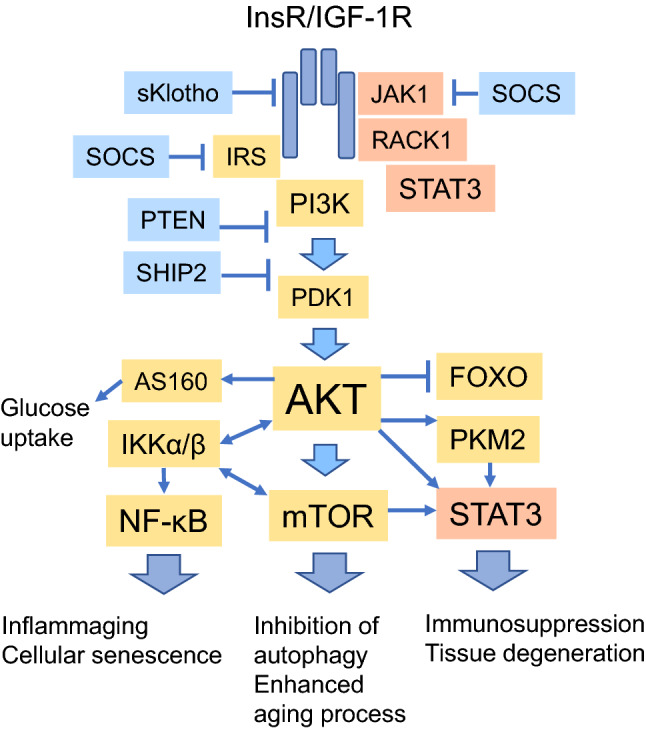
The insulin/IGF-1 signaling pathway including the most relevant connections involved in the activation of STAT3 signaling. The pathway can be inhibited by SOCS, sKlotho, PTEN, and SHIP2 factors. STAT3 signaling can be activated by different pathways; (i) JAK/STAT3/RACK1, (ii) AKT/STAT3, (iii) AKT/PKM2/STAT3, and (iv) AKT/mTOR/STAT3 signaling. AKT signaling activates mTOR kinase and NF-κB signaling, whereas, AKT inhibits the function of FOXO factors. mTOR kinase and the transcription factors STAT3 and NF-κB are associated with the aging process. Moreover, AKT stimulates glucose uptake by inducing the expression of AS160 protein. *AKT* protein kinase B, *AS160* Akt substrate of 160 kDa, *FOXO* Forkhead box O, *IGF-1R* insulin-like growth factor-1 receptor, *IKKα/β* inhibitor of nuclear factor κB kinase α/β, *InsR* insulin receptor, *IRS* insulin receptor substrate, *JAK1* Janus kinase 1, *mTOR* mechanistic target of rapamycin, *NF-κB* nuclear factor κB, *PDK1* 3-phosphoinositide-dependent kinase 1, *PI3K* phosphoinositide 3-kinase, *PKM2* pyruvate kinase isoenzyme M2, *PTEN* phosphatase and tensin homolog, *RACK1* receptor for activated C kinase 1, *SHIP2* SH2-domain containing phosphatidylinositol-3-4-5-trisphosphate 5-phosphatase 2, *sKlotho* soluble Klotho, *SOCS* suppressor of cytokine signaling, *STAT3* signal transducer and activator of transcription 3

Several aging studies have revealed that the insulin/IGF-1 signaling pathway controls the lifespan of both *Caenorhabditis elegans* and mammalian species [1, 2]. There is clear evidence that deficiencies in the function of insulin/IGF-1 signaling can extend lifespan, whereas an increased activity in this pathway promotes the aging process. The insulin/IGF-1 pathway has many crucial functions, e.g., it regulates protein synthesis and energy metabolism as well as the proliferation and differentiation of insulin/IGF-1-responsive cells. For instance, immune cells are responsive to the regulation of insulin/IGF-1 signaling [3]. With respect to the aging process, insulin/IGF-1 signaling controls the activity of several longevity genes, such as mechanistic target of rapamycin (mTOR) and forkhead box O (FoxO) signaling [4, 5] (Fig. 1). The activation of PI3K/AKT signaling, downstream from the insulin/IGF-1 receptors, stimulates the activity of mTOR which in turn is a potent inhibitor of autophagy (Fig. 1). The activity of autophagy declines with aging and thus an excessive activation of mTOR might accelerate the aging process [5]. However, the inhibition of insulin/IGF-1 signaling impairs cellular glucose uptake and enhances the generation of insulin resistance.

**Fig. 2:**
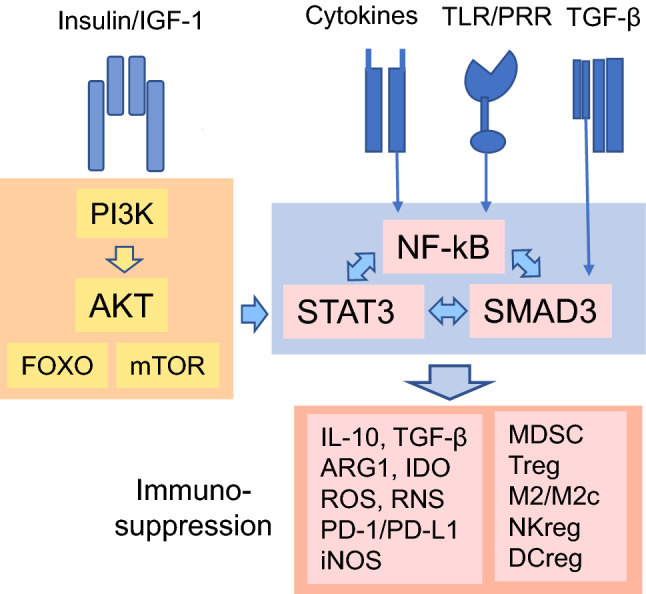
The insulin/IGF-1-induced STAT3 pathway in co-operation with SMAD3 and NF-κB signaling promotes the activation of immunosuppressive cells. These transcription factors induce the expression of several immunosuppressive factors, e.g., immunosuppressive cytokines (IL-10 and TGF-β), enzymes catabolizing amino acids to inhibit cellular proliferation (ARG1 and IDO), reactive oxygen and nitrogen species (ROS, RNS), immune checkpoint proteins (PD-1, PD-L1), and inducible nitric oxide synthase (iNOS). These factors enhance the immunosuppressive phenotype of the immune network including MDSC, Treg, M2/M2c macrophages, NKreg, and DCreg cells. *ARG1* arginase 1, *DCreg* regulatory dendritic cell, *IDO* indoleamine 2,3-dioxygenase, *IL-10* interleukin-10, *iNOS* inducible nitric oxide synthase, *M2/M2c* macrophage M2/M2c phenotype, *MDSC* myeloid-derived suppressor cell, *NKreg* regulatory natural killer phenotype, *PD-1/PD-L1* programmed death-1/programmed death-ligand 1, *PRR* pattern recognition receptor, *ROS* reactive oxygen species, *RNS* reactive nitrogen species, *SMAD3* mothers against decapentaplegic homolog 3, *TGF-β* transforming growth factor-β, *TLR* toll-like receptor, *Treg* regulative T cell phenotype; others are as in Fig. 1

**Fig. 3:**
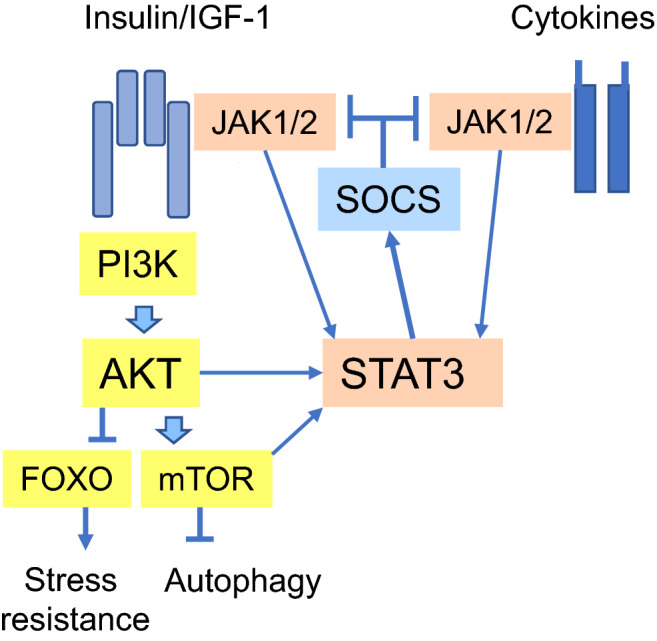
The activation of STAT3 signaling stimulates a negative feedback response through the induction of SOCS factors which inhibit the activity of both insulin/IGF-1 receptors and many cytokine receptors. Accordingly, cytokine receptors can inhibit the activity of insulin/IGF-1 signaling via the STAT3/SOCS signaling and induce insulin resistance. Arrows show the activating responses and stoppers the inhibition of the activity. *AKT* protein kinase B, *AS160* Akt substrate of 160 kDa, *FOXO* Forkhead box O, *IGF-1R* insulin-like growth factor-1 receptor, *IKKα/β* inhibitor of nuclear factor κB kinase α/β, *InsR* insulin receptor, *IRS* insulin receptor substrate, *JAK1* Janus kinase 1, *mTOR* mechanistic target of rapamycin, *NF-κB* nuclear factor κB, *PDK1* 3-phosphoinositide-dependent kinase 1, *PI3K* phosphoinositide 3-kinase, *PKM2* pyruvate kinase isoenzyme M2, *PTEN* phosphatase and tensin homolog, *RACK1* receptor for activated C kinase 1, *SHIP2* SH2-domain containing phosphatidylinositol-3-4-5-trisphosphate 5-phosphatase 2, *sKlotho* soluble Klotho, *SOCS* suppressor of cytokine signaling, *STAT3* signal transducer and activator of transcription 3

The insulin/IGF-1 pathway also regulates the activities of the immune system through different signaling pathways. For instance, there is convincing evidence that insulin/IGF-1 signaling promotes diverse anti-inflammatory/immunosuppressive responses [6] (see below). This is an important function since the aging process is associated with a chronic low-grade inflammation [7]. The signal transducer and activator of transcription 3 (STAT3) protein is a transcription factor known to stimulate immunosuppressive cells, e.g., myeloid-derived suppressor cells (MDSC) and regulatory T cells (Treg), activated in chronic inflammatory conditions [8, 9]. The Janus kinase (JAK)/STAT3 pathway as well as the activation of STAT3 through AKT kinase and mTOR are the important insulin/IGF-1-stimulated, STAT3-mediated signaling pathways which are able to control immunosuppression, immunosenescence, and insulin resistance (Figs. 1, 2, 3). STAT3 signaling also triggers a negative feedback response to the exposure of insulin/IGF-1 and cytokine factors by inducing the expression of suppressor of cytokine signaling proteins (SOCS) (Fig. 3). STAT3 signaling not only inhibit inflammatory responses but also provokes cellular senescence via the STAT3/SOCS/p53 pathway [10]. Given that cellular senescence and immunosuppression both increase with aging, this might explain the acceleration of the aging process through the activation of insulin/IGF-1 signaling. We will examine in detail the activation of immunosuppressive STAT3 signaling induced by the insulin/IGF-1 pathway. Furthermore, we will elucidate the STAT3-mediated processes associated with the inflammaging process and age-related diseases.

## Insulin/IGF-1 paradox of the aging process

Currently, it is known that several signaling networks co-operate in the regulation of the aging process. Interestingly, the insulin/IGF-1 pathway displays an antagonistic pleiotropy with aging, i.e., during development insulin/IGF-1 signaling has fundamental functions in cellular differentiation and the growth of animals, whereas later in the life, it regulates metabolism but its activity clearly accelerates the aging process [1, 2]. Given that growth hormone (GH) controls the level of IGF-1 in the circulation, the GH/insulin/IGF-1 pathway has been called the somatotropic axis in the regulation of mammalian longevity [2]. In particular, there are several mouse models with loss-of-function mutations in signaling through the GH/IGF-1 pathway which have revealed an extension in the animal’s lifespan. For instance, the Ames and Snell mice, which are deficient for GH signaling, and the little mice with a mutation in the growth hormone-releasing hormone receptor (*GHRHR*) gene, are typical long-lived mouse models. These mice display a reduced level of GH/IGF-1 hormones and have a low stature, thus they are called dwarfs. Interestingly, these dwarf mice live 20–70% longer than their wild-type control counterparts. Accordingly, Ortega-Molina et al. [11] reported that transgenic mice overexpressing the phosphatase and tensin homolog (PTEN) protein exhibited a significant extension in their lifespan and a protection against cancers. PTEN phosphatase inhibits the activity of PI3K and thus impedes signaling through the insulin/IGF-1 pathway (Fig. 1). In addition, an overexpression of soluble Klotho (sKlotho) protein which inhibits the function of IGF-1 receptor (IGF-1R) (Fig. 1) extended the lifespan of mice [12]. In humans, the Laron syndrome, caused by mutations in the *GH-R* gene leading to the deficiency in the level of IGF-1, is characterized by obesity, resistance against tumors, and extended lifespan [13]. Moreover, there are studies indicating that experimental treatments which reduce the concentration of IGF-1 in plasma, e.g., caloric restriction, are able to extend the lifespan of several animal species. The attenuation of insulin/IGF-1 signaling is not only associated with increased lifespan but there is convincing evidence that it can alleviate many age-related diseases, such as cancers, cardiovascular diseases, osteoporosis, and neurodegenerative diseases. For instance, Ock et al. [14] demonstrated that the deletion of IGF-1R in mouse cardiomyocytes attenuated the age-related myocardial inflammation, hypertrophy, and interstitial fibrosis. It seems that the antagonistic pleiotropy of insulin/IGF-1 signaling has profound effects on both the healthspan and lifespan of mammalian species.

The insulin/IGF-1 signaling pathway seems to be an evolutionarily well-conserved mechanism involved in the regulation of longevity in species ranging from metazoans to mammals [1, 2]. In *Caenorhabditis elegans*, the Daf-2 pathway, an ortholog of the mammalian insulin/IGF-1 signaling, also displays the antagonistic pleiotropy enhancing the aging process. The loss-of-function mutations of several *daf* (DAuer Formation) genes, e.g., *daf-2* (InsR/IGF-1R), *age-1* (PI3K), and *daf-16* (FoxO), significantly extend the lifespan of *C. elegans* and can induce the dauer phenotype [1, 4, 15]. Accordingly, the expression of Daf-18 protein (ortholog to PTEN), an inhibitor of the Daf-2 signaling pathway, enhances the longevity in *C. elegans* [16]. Moreover, Solari et al. [17] demonstrated that the substitution of the *daf-18* gene with the human *Pten* gene induced a long-lived mutant phenotype of *C. elegans*. It seems that the Daf-16/FoxO transcription factor is the major enhancer of the aging process in *C. elegans*, whereas mTOR (CeTor/Let 363 in *C. elegans*) is a potent promoter of the aging process in rodents [4, 5, 15]. Nonetheless, treatment with rapamycin, a recognized inhibitor of mTOR, induced only a mild extension of lifespan in mice (9% for male and 14% for females) [18]. In another study, a high dose of rapamycin was able to extend the lifespan of mice by 24% (males) and 26% (females) [19]. It is known that an inhibition of mTOR increases autophagy and improves the survival of cells which might extend lifespan [5]. Interestingly, Daf-16/FoxO and mTOR factors have significant immunoregulatory effects via the STAT3 signaling pathways (see below).

## Insulin/IGF-1 signaling promotes immunosuppression

There is convincing evidence that insulin/IGF-1 signaling inhibits inflammatory responses not only by reducing pro-inflammatory hyperglycemia but also by modulating the immune system and decreasing the generation of inflammatory mediators [6]. However, there are studies indicating that under certain conditions insulin/IGF-1 signaling can provoke pro-inflammatory effects, e.g., through disturbing the Th17/Treg balance in autoimmune diseases [20]. This is not surprising since the insulin/IGF-1 pathway is able to control immune responses through a complex signaling network in a context-dependent manner including the activation of the NF-κB system and mTOR signaling. Nonetheless, insulin/IGF signaling has been demonstrated to attenuate inflammatory responses, e.g., in human myocardial infarction [21] and severe thermal trauma [22] as well as in many mouse models, such as colitis [23], vascular inflammation [24], IL-1β-induced cartilage degradation [25], lipopolysaccharide-provoked brain injuries [26], and experimental multiple sclerosis and autoimmune diabetes [27, 28]. Moreover, the anti-inflammatory effects of insulin/IGF-1 therapy in organ transplantation and wound healing might be caused by an expansion of immunosuppressive cells, such as MDSCs and Tregs [29, 30].

Insulin/IGF-1 signaling controls the phenotype and functional activities of immune cells. Ge et al. [23] reported that IGF-1 exposure robustly increased the expression of IL-10, an important immunosuppressive cytokine, in mouse cultured monocytes indicating that IGF-1 enhanced their immune suppressive properties. The IL-10-positive monocytes effectively inhibited the proliferation of CD4^+^ effector T cells in vitro and the adoptive transfer of IL-10-primed monocytes into mice suppressed the inflammatory state in experimental colitis. There are several studies indicating that IGF-1 treatment promotes the polarization of the pro-inflammatory M1 macrophages toward the anti-inflammatory M2 macrophages [31, 32]. Spadaro et al. [32] reported that the IGF-1R-mediated signaling maintained the M2 phenotype of macrophages and that a depletion of IGF-1R from the myeloid cells predisposed mice to the obesity induced by consumption of a high-fat diet. Accordingly, Viardot et al. [33] demonstrated that insulin treatment promoted the differentiation of human Th cells toward an anti-inflammatory Th2 phenotype. This insulin-induced alteration in Th cell polarity might contribute to the immunosuppressive state in chronic inflammatory conditions associated with sepsis, obesity, and type 2 diabetes. There is substantial evidence that insulin/IGF-1 signaling stimulates the expansion of Tregs and increases their immunosuppressive activity [28, 34]. Bilbao et al. [28] demonstrated that IGF-1 exposure stimulated the expansion of human and mouse Treg cells but not that of other T cell phenotypes. The specific deletion of IGF-1R from Treg cells abolished their ability to undergo IGF-1-induced proliferation and the therapeutic effects on experimental mouse encephalomyelitis. Moreover, Johannesson et al. [34] revealed that the ectopic expression of IGF-1 in mouse skin increased the FoxP3-positive Treg cells which secreted an increased level of the IL-10 cytokine. An overexpression of IGF-1 reduced the severity of allergic contact dermatitis in mice. The ablation of IGF-1R from Treg cells eliminated the IGF-1-induced therapeutic response in mouse dermatitis. Currently, the mechanisms of the insulin/IGF-1-evoked activation of immunosuppressive Tregs need to be revealed. It is known that insulin/AKT signaling augments the responsiveness of cells to TGF-β exposure by increasing the trafficking of TGF-β receptors to the cell surface as demonstrated in mouse embryonic fibroblasts and epithelial cells [35]. TGF-β is a potent enhancer of immunosuppression and a co-operative modulator of the immunosuppressive network [36]. Whether or not this sensitization occurs in immune cells still needs to be clarified.

## Insulin/IGF-1 signaling: multiple STAT3-related downstream pathways

### InsR/IGF-1R activate JAK-STAT3 signaling

The InsR and IGF-1R proteins are receptor tyrosine kinases (RTK) and thus they are members of the tyrosine kinase superfamily. The receptors of several growth factors, cytokines, and hormones are RTKs which bind to and phosphorylate different signaling transducers, e.g., members of the JAK family [37]. JAKs activate the transcription factors of the STAT family which have a crucial role in cellular immunity, differentiation, and proliferation. Gual et al. [38] reported that the activation of InsR and IGF-1R induced the binding and phosphorylation of JAK1 and JAK2 proteins, thus promoting their activation in NIH3T3 fibroblasts. Subsequently, JAKs phosphorylated Insulin receptor substrate 1 (IRS1) and 2 (IRS2) which evoked signaling through the PI3K/AKT pathway (Fig. 1). Interestingly, Zong et al. [39] demonstrated that the IGF-1R-induced activation of STAT3 was dependent on the phosphorylation of JAK1 (Fig. 1). Activated JAK1 specifically phosphorylated STAT3 but not the STAT5 factor. They also reported that IGF-1 exposure induced the phosphorylation of the STAT3 factor in several mouse tissues. Accordingly, Coffer et al. [40] reported that InsR specifically phosphorylated STAT3 factor and stimulated its transactivating capacity in a manner which was independent of p21Ras and MAPK signaling, i.e., the other pathways activated by InsR/IGF-1R signaling. Zong et al. [39] revealed that the phosphorylation of STAT3 by JAK1 was prevented by SOCS1 protein. There are other studies indicating that SOCS1 and SOCS6 proteins inhibited the signaling of InsR [41], whereas SOCS2 and SOCS3 proteins suppressed that of IGF-1R [42, 43] (Figs. 1, 3). Moreover, Rui et al. [44] reported that SOCS1 and SOCS3 inhibited the signaling of insulin receptor by inducing a ubiquitin-mediated degradation of IRS1 and IRS2 proteins (Fig. 1). In addition, Kurosu et al. [12] demonstrated that sKlotho inhibited the intracellular signaling of insulin/IGF-1 by preventing the phosphorylation of InsR/IGF-1R in rat hepatocytes (Fig. 1). These studies indicate that the function of the insulin/IGF-1 pathway is abundantly regulated at the receptor level.

Furthermore, Zhang et al. [45] demonstrated that the receptor for activated C kinase 1 (RACK1) protein interacted with InsR/IGF-1R and STAT3, both in in vitro and in vivo conditions, and this interaction was required for the activation of STAT3. RACK1 is a scaffold protein which has a number of binding partners and it is a platform for many signaling processes. It is also known that JAK1 also interacts with RACK1 protein [46]. It seems that RACK1 has a crucial anchoring role in the targeting of JAK1 and STAT3 into the InsR/IGF-1R complex and subsequently, JAK1 can induce the phosphorylation of the STAT3 factor. It is probable that the RACK1 scaffold protein is also involved in the trafficking of STAT3 into different cellular compartments. Interestingly, TGF-β1, a major anti-inflammatory cytokine, is a potent inducer of the expression of RACK1 protein in mouse liver [47]. RACK1 can inhibit NF-κB signaling by binding to the IKK complex and thus it prevents the TNF-α-induced inflammatory response in mouse macrophages [48]. Kiely et al. [49] demonstrated that an overexpression of RACK1 enhanced the activity of IGF-1R kinase, whereas, the IGF-1R-induced activation of PI3K/AKT signaling was inhibited in mouse fibroblasts and MCF-7 cells. This implies that RACK1 enhances the signaling of JAK/STAT3 pathway rather than that of PI3K/AKT (Fig. 1). It seems that an overexpression of RACK1 might enhance immunosuppression by inhibiting NF-κB signaling while at the same time stimulating the insulin/IGF-1-mediated JAK-STAT3 signaling.

### PI3K/AKT activate STAT3 signaling

The PI3K/AKT axis, downstream from the InsR/IGF-1R complex, has several upstream and downstream signaling connections (Fig. 1). For instance, AKT kinase phosphorylates the Akt substrate of 160 kDa (AS160) which triggers the translocation of the glucose transporter GLUT4 to the cell surface, thus enhancing glucose uptake [50] (Fig. 1). Moreover, AKT kinase controls many important signaling pathways, including mTOR, FoxO, IKK, STAT3, and PKM2 (Figs. 1, 2). There is substantial evidence indicating that AKT signaling is able to phosphorylate STAT3 protein although sometimes there is no specification on whether AKT directly phosphorylated STAT3 or whether the activation was induced indirectly through the activation of mTOR or PKM2 (see below). On the other hand, the activation of STAT3 induced the expression of IGF-1R [51] indicating that the cytokine-mediated JAK/STAT3 pathway was able to sensitize insulin/IGF-1 signaling. Moreover, STAT3 can induce the expression of phosphoinositide-dependent kinase-1 (PDK1) [52] which is able to enhance insulin/IGF-1 signaling (Fig. 1). Given that PI3K is an important hub in the function of insulin/IGF-1-dependent regulation, this kinase has been inhibited by PTEN phosphatase (see above) and SH2-containing inositol 5’-phosphatase 2 (SHIP2) which is enriched in mouse brain and its overexpression impaired memory formation [53] (Fig. 1).

Pyruvate kinase isozyme M2 (PKM2) is one of the enzymes which are activated by the IGF-1/AKT axis [54, 55] (Fig. 1). Salani et al. [54] demonstrated that IGF-1 exposure induced the Ser/Thr phosphorylation of PKM2 through the activation of AKT in a human lung cancer cell line (Calu-1). PKM2 controls the last step of glycolysis from phosphoenolpyruvate to pyruvate. This reaction generates ATP without the need for the presence of oxygen and thus the expression of PKM2 is increased in many cancer cells. PKM2 is expressed not only in skeletal muscles but in many other tissues and immune cells, such as monocytes (Human Protein Atlas). PKM2 acts as a glycolytic enzyme in cytoplasm but in nuclei, it can phosphorylate STAT3 protein and enhance STAT3-dependent gene transactivation [56]. Hu et al. [57] reported that 2-deoxyglucose (2-DG), a glycolytic inhibitor and a caloric restriction mimetic, suppressed the LPS-induced nuclear accumulation of PKM2 and the phosphorylation of STAT3. Recently, Hou et al. [58] demonstrated that the sumoylated PKM2 was sorted into ectosomes and subsequently transferred into neighboring cells. This ectosomal PKM2 induced the activation of STAT3 and evoked the differentiation of monocytes into the immunosuppressive M2 macrophages. The IGF-1/AKT/PKM2 axis not only controls energy metabolism but it can regulate gene expression and tissue homeostasis through STAT3 signaling.

### AKT/mTOR activate STAT3 signaling

The complexes of mechanistic target of rapamycin (mTOR), i.e., mTORC1 and mTORC2, are important downstream targets of PI3K/AKT signaling (Fig. 1). Saxton and Sabatini [59] have reviewed in detail the activation steps of the mTOR signaling network. mTOR kinase has a key role not only in the control of protein synthesis, autophagy, and metabolism but also in the regulation of immune responses and the aging process. mTOR kinase phosphorylates STAT3 protein at Tyr705 and Ser727 which are typical phosphorylation sites of STAT3 [60, 61]. Accordingly, the mTOR/STAT3 pathway controls several important downstream targets, such as HIF-1α and Notch1, which are involved in the regulation of insulin resistance [61–63]. Moreover, mTOR/STAT3 signaling is a crucial pathway in the generation of the immunosuppressive state driven by STAT3 (see below). There are several inhibitors of mTOR kinase, both physiological and pharmacological molecules, which are able to control both insulin resistance and immunosuppression. Especially, AMPK is a potent inhibitor of the mTOR-mediated signaling, e.g., it can inhibit the MDSC-induced immunosuppressive responses, probably inhibiting the mTOR-STAT3 pathway [64]. Moreover, Gadd45a, a p53-regulated and DNA-damage-inducible protein, can suppress the function of mTOR/STAT3 signaling [65]. Gadd45a interacts with mTOR protein and thus suppresses the Ser727 phosphorylation of STAT3. It seems that many effects of AKT/mTOR signaling on the immune system and the aging process are mediated through the activation of STAT3 signaling.

Rapamycin and the recently developed more specific mTOR inhibitors, i.e., rapalogs, are promising therapeutic compounds in the treatment of cancers and several autoimmune and chronic inflammatory diseases [66]. There is an active debate about whether rapamycin and other rapalogs can extend human healthspan and lifespan. Although the inhibition of mTOR stimulates autophagy, these therapeutic observations are somewhat surprising since mTOR signaling is the master regulator of many survival processes and thus its inhibition should evoke harmful responses. It is known that mTOR signaling is robustly upregulated in conditions of chronic inflammation and in these situations, rapamycin therapy seems to be efficient [66].

### AKT/FOXO control STAT3 signaling

Forkhead class O transcription factors (FoxO), especially FoxO1 and FoxO3a, have crucial functions in T cell biology, e.g., they regulate the differentiation and survival of T cells and the development of immunosuppressive Tregs [67]. For instance, FoxO factors induce the expression of FoxP3 transcription factor which determines the ultimate properties of the Treg cell lineage [68]. FoxO factors can also extend the lifespan in *C. elegans*, as seen earlier, although in mammals, the role of FoxOs needs to be clarified. Kyoung Kim et al. [69] demonstrated that the knockdown of FoxO3a enhanced cellular senescence in human dermal fibroblasts indicating that FoxO3a might maintain cellular homeostasis, probably by enhancing autophagy. It is known that FoxO proteins are important downstream targets of the AKT-induced phosphorylation (Fig. 1). The AKT-induced phosphorylation of FoxO proteins generates the binding sites for the 14-3-3 proteins which induce the accumulation of FoxO proteins into the cytoplasm thus preventing their transcriptional activity. Interestingly, Oh et al. [70] demonstrated that the phosphorylated STAT3 proteins interacted with the phosphorylated FoxO1 and FoxO3a proteins in the cytoplasm of mouse CD4^+^ T cells and subsequently promoted the import of the complex into nuclei where the dephosphorylated FoxO proteins transactivated specific genes. The loss of STAT3 proteins caused the nuclear exclusion of FoxO proteins. The nuclear translocation of activated AKT induced the phosphorylation of FoxO proteins which evoked the 14-3-3-mediated export of FoxO proteins from the nuclei. Oh et al. [71] also reported that the activation of STAT3 enhanced the FoxO-dependent expression of inhibitor of κB (IκB) proteins which induced the accumulation of NF-κB complexes into cytoplasm, inhibiting the pro-inflammatory NF-κB-dependent gene expression. This indicates that STAT3 proteins can control the function of pro-inflammatory NF-κB through an activation of FoxO proteins.

### AKT/mTOR/IKK activate NF-κB signaling

The NF-κB system is the hub of upstream and downstream signaling connections which regulate innate and adaptive immunity as well as the functions associated with cell growth and death. The NF-κB system acts in close co-operation with several signaling pathways, e.g., those of JAK-STAT3, SMAD3, and insulin/IGF-1 signaling (Fig. 2). There is substantial evidence that the InsR/IGF-1R axis can activate NF-κB signaling in both immune and non-immune cells [72–74] (Fig. 1). Commonly, this activation of the NF-κB system is mediated via the PI3K/AKT/IKKα/β pathway although it is known that InsR/IGF-1R can also activate the NF-κB complex through the RAF-1 [72] and MAPK p38 [75] pathways. Moreover, there are specific cell-type differences in the expression of either IKKα or IKKβ, i.e., the activation of IKKα is more dependent on the signaling of PI3K/AKT than that of IKKβ [74]. Gustin et al. [76] demonstrated that AKT activated IKKα which induced the non-canonical NF-κB signaling by processing the NF-κB2 (p100) protein to the p52 component of NF-κB in HEK293 cells. The activation of the non-canonical NF-κB pathway has an important role in the functions of adaptive immunity. We have earlier discussed in detail the role of the insulin/IGF-1-driven AKT/IKK/NF-κB signaling pathway in the aging process [77].

There is close crosstalk between IKKα/β and mTOR in the activation of NF-κB signaling (Fig. 1). IKKα/β are specific activators of NF-κB signaling although IKKα can also activate both the AKT and mTOR kinases [78–80]. Dan and Baldwin [81] revealed that insulin and TNFα activated mTOR through both the AKT/mTOR and the AKT/IKKα/mTOR pathways, whereas TNFα also activated mTOR via the IKKβ-mediated signaling independently of the PI3K/AKT pathway in several cell types. Interestingly, Dan et al. [78] demonstrated that treatment of PC3 cells with rapamycin downregulated the activity of IKKα and inhibited the expression of many NF-κB-dependent genes. This is an interesting observation indicating that the exposure to rapamycin not only inhibited mTOR activity but also prevented the IKKα-dependent activity of the NF-κB system. Even more intriguing are observations which revealed that NF-κB and STAT3 factors can form complexes with each other to activate or repress the transcription of target genes either synergistically or through κB or STAT3 binding motifs [82, 83]. This context-dependent cooperation still needs to be clarified in different immune cells.

## JAK-STAT3 signaling: a master regulator of immunosuppression

### Overview on the JAK-STAT3 signaling pathway

The JAK-STAT3 pathway transduces signals from the cell surface receptors of distinct cytokines, growth factors, and hormones into the nuclei. For instance, the receptors of IL-6, IL-10, IL-23, interferons, EGF, and Ins/IGF-1 as well as certain G-protein coupled receptors (GPCR) are linked to the activation of JAK1/JAK2 which subsequently recruit STAT3 proteins to the receptor complex and phosphorylate STAT3 at Tyr705 [37]. The phosphorylated STAT3 proteins form homo- or heterodimers with other STAT proteins and these dimers are then translocated into nuclei where they transactivate specific target genes. Hutchins et al. [84] characterized the genome-wide DNA-binding sites of STAT3 factors upon the IL-10-induced anti-inflammatory response in mouse macrophages. They observed that the binding sites were specifically associated with the genes controlling immune functions and furthermore, the binding positively regulated the expression of these genes. Moreover, STAT3 proteins can form complexes with other transcription factors, e.g., FoxO factors, NF-κB complexes as well as SMAD3 proteins [85] (Fig. 2), which affect their nuclear translocation and binding to the promoters of specific genes. The JAK-STAT3 signaling pathway controls its own activity by inducing the expression of SOCS proteins, as described above. The dephosphorylation of STAT3 by different protein phosphatases inhibits the signaling through the JAK-STAT3 pathway. There are several protein tyrosine phosphatases (PTP) of receptor type, e.g., the PTP receptor type T (PTPRT), which can specifically dephosphorylate the pTyr705 amino acid of STAT3 [86]. Two cytoplasmic PTPs, i.e., the Src-homology 2 domain (SH2)-containing SHP-1 and SHP-2, can also target the Tyr705 of STAT3 [87]. In addition, T-cell PTP (TCPTP) dephosphorylates STAT3 and thus attenuates insulin signaling in mouse liver [88]. Moreover, the expression and phosphorylation status of JAKs and STAT3 can be controlled by several microRNAs in a context-dependent manner. The JAK-STAT3 signaling pathway not only has a crucial role in the regulation of innate and adaptive immunity but in non-immune cells, it can control cell proliferation, metabolism, and cellular senescence in a context-dependent manner (see below).

### STAT3 signaling controls the activity of immunosuppressive cells

There is convincing evidence that STAT3 signaling has a crucial role in the generation of an immunosuppressive microenvironment around tumor sites [89]. STAT3 is an important oncoprotein which is activated in most tumors in which it stimulates the secretion of anti-inflammatory factors, such as IL-6, IL-10, IL-23, GM-CSF, and TGF-β. STAT3 signaling is a major inducer of the differentiation of myeloid cells toward immunosuppressive phenotype in tumors [89]. In fact, it is not only tumor microenvironments which can educate immune cells since for instance, the GM-CSF can license murine and human monocytes toward their immunosuppressive phenotypes through the PI3K/AKT/mTOR signaling pathway [90]. Senescent cells secrete increased levels of colony-stimulating factors, chemokines, and cytokines, e.g., GM-CSF, IL-6, IL-10, and TGF-β, which can recruit and educate immune cells in aged tissues (see below). For instance, Chiu et al. [91] demonstrated that the host tissue microenvironment was responsible for the age-related functional deficiency of natural killer (NK) cells in mice. Next, we will shortly examine the role of STAT3 signaling in the generation of immunosuppressive phenotype of distinct immune cells known to be affected by the aging process.

The immunosuppressive network refers to the immune cells which express the regulatory, immune suppressive activities; these cells include MDSCs, Tregs, Bregs, M2 macrophages (Mreg), and the regulatory subsets of dendritic cells (DCreg), NK cells (NKreg), and type II natural killer T cells (NKT) [92]. This is a cooperative network which not only enhances their own immunosuppressive properties but it also suppresses the functions of pro-inflammatory effector immune cells. The immunosuppressive network acts via the secretion of anti-inflammatory cytokines, such as IL-10, TGF-β, IL-4, IL-27, IL-35, as well as the release of reactive oxygen and nitrogen species (ROS/RNS). Immunosuppressive cells also express arginase 1 (ARG1) and indoleamine 2,3-dioxygenase (IDO) which deplete levels of L-arginine and tryptophan, respectively, from the microenvironment and thus suppress the functions of effector immune cells [92] (Fig. 2). In addition, immunosuppressive cells target effector cells through the expression of immune checkpoint proteins, e.g., the PD-1/PD-L1 and CTLA-4 proteins, which suppress the activation of T cells and prevent antigen-presentation. Interestingly, the STAT3 transcription factor is the major inducer of the expression of this immunosuppressive armament, either directly or indirectly [9, 89, 93, 94].

Inflammatory mediators released from peripheral tissues enhance myelopoiesis, especially the generation of immunosuppressive, immature MDSCs, mostly in bone marrow (BM). Zhang et al. [95] reported that the infection-induced granulocyte colony-stimulating factors (G-CSF) stimulated the STAT3-mediated expansion of myeloid progenitor cells in mouse BM. de Koning et al. [96] reported that STAT3 induced the expression of p27KIP1 which stimulated the differentiation of mouse myeloid cells, and moreover, maintained their survival. It is known that several cytokines, such as IL-6, IL-10, and TGF-β, which activate STAT3 signaling, are potent inducers of the differentiation and activation of MDSCs [97]. Interestingly, it is known that insulin/IGF-1 signaling can control the growth of hematopoietic progenitor cells and myeloid differentiation both in *Drosophila* and mammalian species [98–100]. In *Drosophila*, insulin/TOR signaling regulates the size and differentiation of the hematopoietic niche [99]. Duan et al. [100] demonstrated that insulin signaling modulated the differentiation of mesodermal and hematopoietic lineages in human pluripotent stem cells in a stage-specific manner. Insulin/mTOR signaling was crucial for the differentiation of hematopoietic progenitor cells to granulocyte and monocyte/macrophage populations. It seems that insulin can also enhance lymphoid cell differentiation via STAT3 signaling. Xia et al. [101] demonstrated that the insulin/mTOR/STAT3 pathway acted through the transcription of the *Ikaros* gene to induce the commitment of hemotopoietic progenitors to the lymphoid lineage. There is convincing evidence that the aging process affects the differentiation of hematopoietic lineages, i.e., myelopoiesis is clearly upregulated with aging [102].

Immunosuppressive Treg cells have crucial functions in the maintenance of self-tolerance and immune homeostasis in diverse pathological conditions. Tregs not only suppress the functions of effector T cells but they can also inhibit the activities of innate immunity, e.g., that of NK cells [103]. In addition, several immunosuppressive cells, such as MDSCs, DCregs, and Bregs, can convert resting CD4 T cells into immunosuppressive Tregs [104, 105]. This plasticity in the properties of Tregs is largely based on the epigenetic control of the FoxP3 transcription factor which is the major driver of the differentiation and immunosuppressive properties of Tregs [106]. Interestingly, several studies have revealed that STAT3 signaling increases the expression of FoxP3 and thus enhances the immunosuppressive activity of Tregs [8, 107]. It is known that STAT3 signaling can modulate the chromatin landscape associated with the expression of the FoxP3-driven genes. Kim et al. [108] demonstrated that JAK2 phosphorylated lysine-specific demethylase 3A (KDM3A) which subsequently acted as a STAT3-dependent transcriptional coactivator. Hossain et al. [109] demonstrated that FoxP3 factor formed complexes with STAT3 protein which consequently transactivated the expression of the *IL-10* gene in human breast cancer-associated Treg cells. There is a robust evidence that IGF-1 stimulates the proliferation and increases the immunosuppressive activity of FoxP3-positive Tregs in mouse and human cells [27, 28, 110]. Bilbao et al. [28] demonstrated that recombinant human IGF-1 (rhIGF-1) increased the expression of FoxP3 factor and in vitro stimulated the proliferation of murine Tregs but not that of other T-cell subtypes. Systemic administration of rhIGF-1 suppressed the progression of mouse autoimmune disease models, i.e., type 1 diabetes and multiple sclerosis. The ablation of IGF-1 receptor from Tregs abrogated the beneficial effects of rhIGF-1. Moreover, Dedovic et al. [111] revealed the presence and enrichment of insulin-specific, FoxP3-positive Treg cells in mouse lymphoid tissues. These studies indicate that both insulin/IGF-1 and STAT3 signaling have fundamental roles in the differentiation of immunosuppressive phenotype of Tregs although the pathways need to be clarified in more detail.

Macrophages and NK cells are major players in the maintenance of innate immune defense. There is substantial evidence that the STAT3-dependent signaling induces the polarization of macrophages toward the immunosuppressive M2 phenotype [112]. Interestingly, Nakamura et al. [113] demonstrated that in the eyes of aged mice, the increased signaling of the STAT3 pathway induced the M2 polarization of macrophages, thus enhancing vascular proliferation and subsequently the formation of angiogenesis. They also reported that the expression of SOCS3 protein was clearly reduced in senescent M2 macrophages which probably aggravated STAT3 signaling. There are also studies indicating that insulin can promote the macrophage transition from the pro-inflammatory M1 phenotype to the anti-inflammatory M2 phenotype through the PI3K/AKT signaling pathway [114, 115]. Accordingly, Cacalano [116] has described in detail the role of STAT3 signaling in the development, activation, and cytotoxicity of NK cells. In brief, Burgess et al. [117] reported that the phosphorylation of STAT3 on Tyr705 and Ser727 clearly decreased the expression of activating natural killer group 2, member D (NKG2D) recognition receptor and its adapter protein DAP10, thus reducing the NKG2D-mediated cytotoxic activity of human primary NK cells. This change impaired the NKG2D-dependent immune surveillance of tumor cells and probably also that of senescent cells which robustly express NKG2D ligand proteins [118]. The deletion of the *Stat3* gene increased the level of two cytolytic enzymes perforin and granzyme B in mouse NK cells and accordingly a lack of STAT3 signaling enhanced the NK cell-dependent tumor surveillance [119]. It is known that the activation of STAT3 signaling in the cells of host tissues produces soluble factors which are able to repress both the migration and the recruitment of NK cells, e.g., into pro-inflammatory tumor sites [116]. Moreover, the STAT3-activated immunosuppressive cells, e.g., MDSCs and Tregs, can directly inhibit the cytotoxic activity of NK cells [120, 121]. It seems that the STAT3-induced expansion of immunosuppressive cells inhibits the surveillance of senescent cells which enhances their accumulation within tissues with aging [122].

### Crosstalk between STAT3, NF-κB, and SMAD3 signaling

STAT3, NF-κB, and SMAD3 transcription factors are the main regulators of immune functions involving both the differentiation of immune cells and the modification of their different phenotypes and properties. The crosstalk between these factors is mediated through their mutual interaction or their abilities to control the expression of compounds which affect the coordination of their activities (Fig. 2). For instance, the receptors of the TGF-β superfamily are able to activate either SMAD3 or STAT3 signaling in a context-dependent manner [123]. Wang et al. [85] reported that phosphorylated STAT3 selectively interacted with SMAD3 protein, thus blocking the formation of transactivating SMAD3/SMAD4 complex in cultured human liver cells and keratinocytes. These studies highlight that the phosphorylation of STAT3 can inhibit the SMAD3-dependent gene expression. On the other hand, Bryson et al. [124] observed that the phosphorylation of STAT3 through Oncostatin M signaling promoted the formation of a complex between pSTAT3 and SMAD3 in the cytoplasm of human HMEC cells. Subsequently, the STAT3/SMAD3 complex was translocated into nuclei where the SMAD3-dependent transcription induced the cellular senescence of HMEC cells. In addition, pSTAT3 can interact with FoxO1 and FoxO3 in cytoplasm and translocate these factors into nuclei where they transactivate the FoxO-dependent genes (see above). These studies indicate that the activation of STAT3 through different pathways of insulin/IGF-1 signaling is not only able to stimulate the expression of STAT3-dependent genes but it also affects the expression profiles of other genes, e.g., those activated by SMAD3 and FOXO factors.

There is also an active co-operation between STAT3 and NF-κB signaling driving either pro-inflammatory or anti-inflammatory responses in a context-dependent manner. As described earlier, the connections between AKT/mTOR/IKK signaling hub is able to control the activity of NF-κB signaling through different pathways (Fig. 2). Interestingly, Yang et al. [82] reported that unphosphorylated STAT3 (U-STAT3) could bind to several promoters which do not contain the binding site for pSTAT3, e.g., *IL-6* and *RANTES* genes, in human HME1 cells. They revealed that U-STAT3 interacted with the NF-κB complex in cytoplasm, competing with the binding of IκB proteins which are specific inhibitors of NF-κB activation. The U-STAT3/NF-κB complex translocated into nuclei exploiting the nuclear localizing sequence (NLS) of STAT3 protein and targeted to a subset of NF-κB-dependent genes. Lee et al. [83] reported that a persistent stimulation of STAT3 facilitated the signaling of NF-κB by enhancing the acetylation of the RelA component in human melanoma and prostate cancer cells. The activation of STAT3 signaling stimulated the expression of p300 acetyltransferase which acetylated RelA protein and thus potentiated the expression of distinct NF-κB-driven genes. This means that the activation of STAT3 signaling, e.g., through the insulin/IGF-1 pathway, is able to stimulate pro-inflammatory/pro-tumorigenic signaling in the host tissue. On the other hand, the activation of NF-κB signaling can enhance the STAT3-dependent immunosuppression. For instance, Martincuks et al. [125] revealed that the stimulation of NF-κB signaling increased the expression of STAT3 protein, but not that of STAT5, in mouse fibroblasts. Accordingly, Yu et al. [126] demonstrated that STAT3 signaling activated the expression of IDO, a major inducer of the immunosuppressive milieu, without the need for binding of STAT3 protein to the promoter of *IDO* gene in human MDSCs. They revealed that STAT3 signaling induced the expression of IDO through the activation of noncanonical NF-κB signaling through the binding of the RelB/p52 complex to the promoter of *IDO* gene. These studies indicate that STAT3 signaling can utilize NF-κB components to evoke an immunosuppressive state in host tissues.

## Immunosuppression, immunosenescence, and insulin resistance associated with inflammaging

### Immunosuppression increases with aging and age-related diseases

Currently, it is known that a low-grade inflammatory process is associated with a compensatory anti-inflammatory state which involves the activation of the immunosuppressive network. Recently, we have reviewed in detail the immunosuppressive remodeling of the immune system in conjunction with aging [92]. In brief, the numbers of MDSCs significantly increase with aging both in the circulation of humans and mice [127, 128]. The quantity of MDSCs is also elevated with aging in mouse bone marrow, spleen, and lymph nodes [127, 129, 130]. It is not only the frequencies of MDSCs but also their immunosuppressive activities clearly increase in immune tissues. There is also a robust increase with aging in the numbers of Tregs in the circulations of both humans and mice [131, 132]. Moreover, the amount of FoxP3-positive Tregs increases with aging in mouse skin and adipose tissues [133, 134]. Aging also expands the amounts of immunosuppressive M2 macrophages in many mouse tissues, e.g., bone marrow, spleen, lungs, and skeletal muscles [135, 136]. The aging process also affects the phenotypes of NK and dendritic cells remodeling them toward immunosuppressive phenotypes [92]. Interestingly, Ruhland et al. [137] revealed that the accumulation of pro-inflammatory senescent stromal cells in mouse skin increased the presence of MDSCs and Tregs in mouse skin. They also reported that the age-related increase in the number of senescent cells in human skin was not only associated with an upregulation of inflammatory factors but also with an elevated level of MDSCs. These results imply that increased cellular senescence with aging maintains a low-grade inflammatory condition which is counteracted by the accumulation of immunosuppressive cells into aging tissues [122].

There are many clinical observations indicating that the aging process is associated with a profound remodeling of the immune system. The inflammaging process involving the accumulation of pro-inflammatory senescent cells triggers immunosuppressive mechanisms to maintain tissue homeostasis [122]. For instance, the risk for cancers and chronic infections increases with aging. Moreover, it is recognized that the efficiency of vaccination and immunotherapy is diminished in elderly people. Ladomersky et al. [138] demonstrated that the expansion of immunosuppressive cells in brain reduced the efficacy of immunotherapy for glioblastoma in elderly patients. In contrast, it has been observed that older people have a lower risk for the rejection of heart transplants [139] which indicates that an increased immunosuppressive state improves their transplantation tolerance. There is clear evidence that several age-related pathologies are associated with an increased presence of immunosuppressive cells. For instance, the progress of atherosclerosis involves an increased number of pro-inflammatory M1 and immunosuppressive M2 macrophages [140] as well as an enhanced accumulation of MDSCs and Tregs [141, 142]. It seems that MDSCs and Tregs exert therapeutic effects since they suppress the pro-inflammatory immune processes and attempt to resolve accumulating plaques. However, in chronic inflammatory conditions, e.g., in inflammaging, where the source/cause cannot be resolved, an increased level of MDSCs, Tregs, and M2 macrophages and the molecular mechanisms which they use to induce immune suppression can impair tissue homeostasis and promote age-related degeneration [143].

### STAT3 signaling controls cellular senescence and immunosenescence

There is clear evidence that insulin/IGF-1 exposure can induce/augment the cellular senescence of different non-immune cells in several experimental conditions [144–147]. It does seem that there are several STAT3-mediated pathways, either insulin/IGF-1-dependent or -independent, which can induce cellular senescence in a context-dependent manner. Zhao et al. [147] reported that IGF-1 exposure induced the activation of AKT kinase which subsequently triggered the p53-dependent senescence in rat articular chondrocytes. Several other investigators have also revealed that the activation of IGF-1R stimulated the p53-dependent cellular senescence, e.g., in human and mouse fibroblasts, human mesangial cells, and rat vascular smooth muscle cells [144–146]. Moreover, exposure to IGF binding protein 5 (IGFBP5), the transport protein of IGF-1, provoked cellular senescence in human umbilical vein endothelial cells (HUVEC) [148] although the signaling pathway remained unknown. STAT3 signaling might have been involved since Kojima et al. [149] demonstrated that STAT3 induced the expression of IGFBP5 which consequently triggered a premature senescence of human fibroblasts. It is known that STAT3/SOCS signaling can promote cellular senescence by activating the p53-induced gene expression [10, 150]. The IGF-1/p53-mediated cellular senescence (see above) might also be induced by the STAT3/SOCS pathway. These examples clearly indicate that the activation of STAT3 signaling is able to trigger cellular senescence, probably in co-operation with NF-κB and SMAD3 signaling.

The aging process is associated with a significant remodeling of both the innate and adaptive immune systems. A chronic low-grade inflammaging process is accompanied by immunosenescence, i.e., a gradual deterioration of the immune system with aging, especially the functions of adaptive immunity decline [7, 151]. Immunosenescence also appears in *Caenorhabditis elegans* and *Drosophila* [152, 153]. Currently, there is a debate about the mechanisms involved in immune deficiency and their role in the aging process. Immunosenescence is characterized by functional deficiencies of the effector type of T, B, NK, and dendritic cells, whereas the regulatory, immunosuppressive phenotypes of these cells increase with aging [122]. The hallmarks of cellular senescence, e.g., the appearance of senescence-associated β-galactosidase (SA-β-gal) and heterochromatin foci (SAHF) as well as the increased expression of cell cycle inhibitor proteins, i.e., p16, p21, and p53, are also common markers of senescent immune cells, especially in Teff cells [154–156]. Interestingly, there are observations that immunosuppressive cells, such as MDSCs and Tregs, not only can induce senescence markers in immune cells but also impair their functional competence [154, 155]. There are many observations that insulin/IGF-1 signaling is not only able to trigger the cellular senescence of non-immune cells but it evokes immunosenescence in both mammals and *C. elegans* [157, 158]. It is known that conditions lowering the level of insulin/IGF-1 factors, e.g., dwarfism and prolonged fasting, improve the function of the immune system with aging [157, 159]. The major target of insulin/IGF-1 signaling might be mTOR and its connection to STAT3 (Fig. 1). Hurez et al. [160] demonstrated that in mice the inhibition of mTOR with rapamycin increased the numbers of effector cells, e.g., naïve T and B cells, and innate lymphoid cell populations, whereas the numbers of exhausted T cells were reduced. Moreover, Mannick et al. [161] reported that the rapalog, RAD001, improved immune functions in elderly people, e.g., it enhanced the response to influenza vaccination. These results indicate that the inhibition of mTOR is able to reduce immunosenescence which might explain the lifespan extension of mice with rapamycin therapy [18, 19].

### Insulin resistance: STAT3/SOCS signaling suppresses insulin/IGF-1 pathway

The signaling pathways of several cytokines and growth factors, such as insulin/IGF-1, converges at the activation of STAT3 factor (Figs. 2, 3) which is the nodal point for both inflammatory and growth factor communication. Interestingly, increased STAT3 signaling exerts a negative feedback response to the function of the JAK-STAT3 signaling pathways, i.e., STAT3 factor induces the expression of SOCS proteins which can interact with and inhibit the function of cytokine receptors and insulin/IGF-1 receptors (Figs. 1, 3). There is substantial evidence that the expression of SOCS3 protein increases with aging in several cells/tissues, such as rat myocardium [162], hypothalamus [163], and human blood mononuclear cells [164] as well as human sarcopenic skeletal muscles [165] and the brains of Alzheimer’s patients [166]. Thus, although age-related low-grade inflammation is a major inducer of STAT3/SOCS3 signaling, it is not only signaling via the cytokine receptors but also that of insulin/IGF-1 receptors which will be inhibited through the signaling of the STAT3/SOCS3 pathway (Fig. 3).

In the mid of 1980’s, it was observed that insulin resistance (IR) was associated with the aging process in human tissues [167, 168]. Currently, it is known that there exist diverse, tissue-specific mechanisms to repress the signaling through the insulin/IGF-1 pathway. Ueki et al. [169] reported that SOCS1 and SOCS3 induced IR by inhibiting the phosphorylation of tyrosine moieties on IRS proteins in mouse adipocytes, muscle cells, and liver tissue. Yadav et al. [170] demonstrated that IGF-1 exposure activated the STAT3-induced expression of SOCS3 protein which subsequently was able to bind to IGF-1R and inhibit the IGF-1-driven JAK1-STAT3 signaling in rat primary cortical neurons. Accordingly, Al-Shanti and Stewart [171] demonstrated that IL-6 exposure, which is known to activate STAT3 signaling, inhibited the activity of IGF-1 and myogenesis in mouse myoblasts. The IR in adipose tissue is a well-known experimental model which has been exploited to clarify the different mechanisms involved in both age-related and obesity-induced IR in rodents. Bapat et al. [134] revealed that different immune cell populations within adipose tissue were associated with the aging- and obesity-induced IR in mice. They observed that the number of Tregs robustly increased with aging in mouse adipose tissue and their depletion prevented the age-related IR in adipose tissue. In contrast, inflammation and the accumulation of macrophages elicited obesity-induced IR in mouse adipose tissue. Currently, it is known that cellular senescence has a key role in the generation of type II diabetes [172]. It seems that increased cellular senescence in peripheral tissues is associated with insulin resistance as well as the functional deficiency of pancreatic β-cells. The activation of JAK/STAT3 signaling, either stimulated by insulin/IGF-1 or cytokine signaling, is able to trigger the STAT3/SOCS/p53 pathway, thus promoting cellular senescence and immunosenescence (see above). It seems that while the negative feedback of the STAT3 signaling delays the insulin/IGF-1-induced aging process, it nonetheless elicits insulin resistance and diabetes.

## Conclusions

The antagonistic pleiotropy in the function of the insulin/IGF-1 pathway reveals a paradox of the aging process since insulin/IGF-1 signaling is an important signaling pathway of growth and differentiation during development but its function accelerates the aging process later in the lifespan. mTOR signaling, a downstream target of the insulin/IGF-1 pathway, is a well-known enhancer of the anabolic processes required during the growth phase when its capacity to inhibit autophagy might be beneficial but later in the lifespan, its inhibition promotes the degeneration of aging tissues. Interestingly, insulin/IGF-1 signaling is also able to control immunosuppression and cellular senescence through its several connections to STAT3 signaling. For instance, STAT3 signaling is a potent enhancer of immunosuppressive properties in MDSCs, Tregs, and M2 macrophages. The increase in the numbers of these cells with aging not only counteracts chronic age-related inflammation but also disturbs the maintenance of tissue homeostasis, enhancing cellular senescence and tissue degeneration [143]. For instance, STAT3 signaling can induce cellular senescence in many cell types through the STAT3/SOCS/p53 pathway. The STAT3 factors induce the expression of SOCS proteins which consequently inhibit the function of JAK/STAT3 signaling, i.e., SOCS proteins exert a negative feedback on the JAK/STAT3 signaling induced by both insulin/IGF-1 and certain cytokine pathways. Given that SOCS proteins inhibit insulin/IGF-1 signaling, they are able to elicit insulin resistance, a condition which is known to be associated with aging. It seems that inflammaging stimulates JAK/STAT3 signaling which augments the expression of SOCS proteins and subsequently promotes cellular senescence and insulin resistance in a cell-type dependent manner. It has been reported that the STAT3 transcription factor can induce epigenetic remodeling of chromatin structures [173]. It is known that cellular senescence and also the aging process are associated with epigenetic remodeling of the chromatin landscape. It seems that STAT3 signaling possesses the properties which might reprogram the phenotypes of both immune and non-immune cells to enhance the aging process and thus this phenomenon may be an important contributor to age-related diseases.
